# Supplementary material for: sCIN: a contrastive learning framework for single-cell multi-omics data integration
Source: Brief Bioinform. 2025 Aug 26;26(4):bbaf411. doi: 10.1093/bib/bbaf411 (PMC12378934; doi:10.1093/bib/bbaf411)
Supplement: Supplementary_bbaf411 [file supplementary_bbaf411.pdf]

sCIN: A Contrastive Learning Framework for single-cell Multi-omics Data Integration

Amir Ebrahimi[1] , Alireza Fotuhi Siahpirani[2], Hesam Montazeri[2*]

[1] Department of Biotechnology, College of Science, University of Tehran, Tehran, Iran

[2] Department of Bioinformatics, Institute of Biochemistry and Biophysics, University of Tehran, Tehran, Iran

* Corresponding author: Hesam Montazeri, Department of Bioinformatics, Institute of Biochemistry and Biophysics, University of Tehran, Tehran, Iran. Email: hesam.montazeri@ut.ac.ir

Supplementary Figures

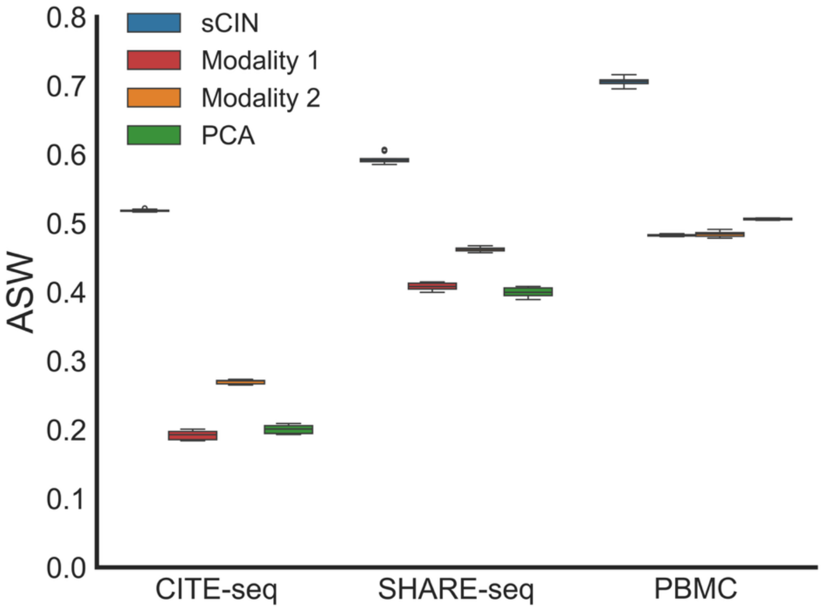

Supplementary Figure 1. Comparison of ASW metric between each original modality, joint PCA embeddings of modalities, and joint sCIN embeddings for each paired dataset. For the SHARE-seq and the PBMC datasets, Modality 1 and Modality 2 are gene expression and chromatin accessibility, respectively. For the CITE-seq dataset, Modality 1 and Modality 2 are gene expression and cell surface proteins, respectively.

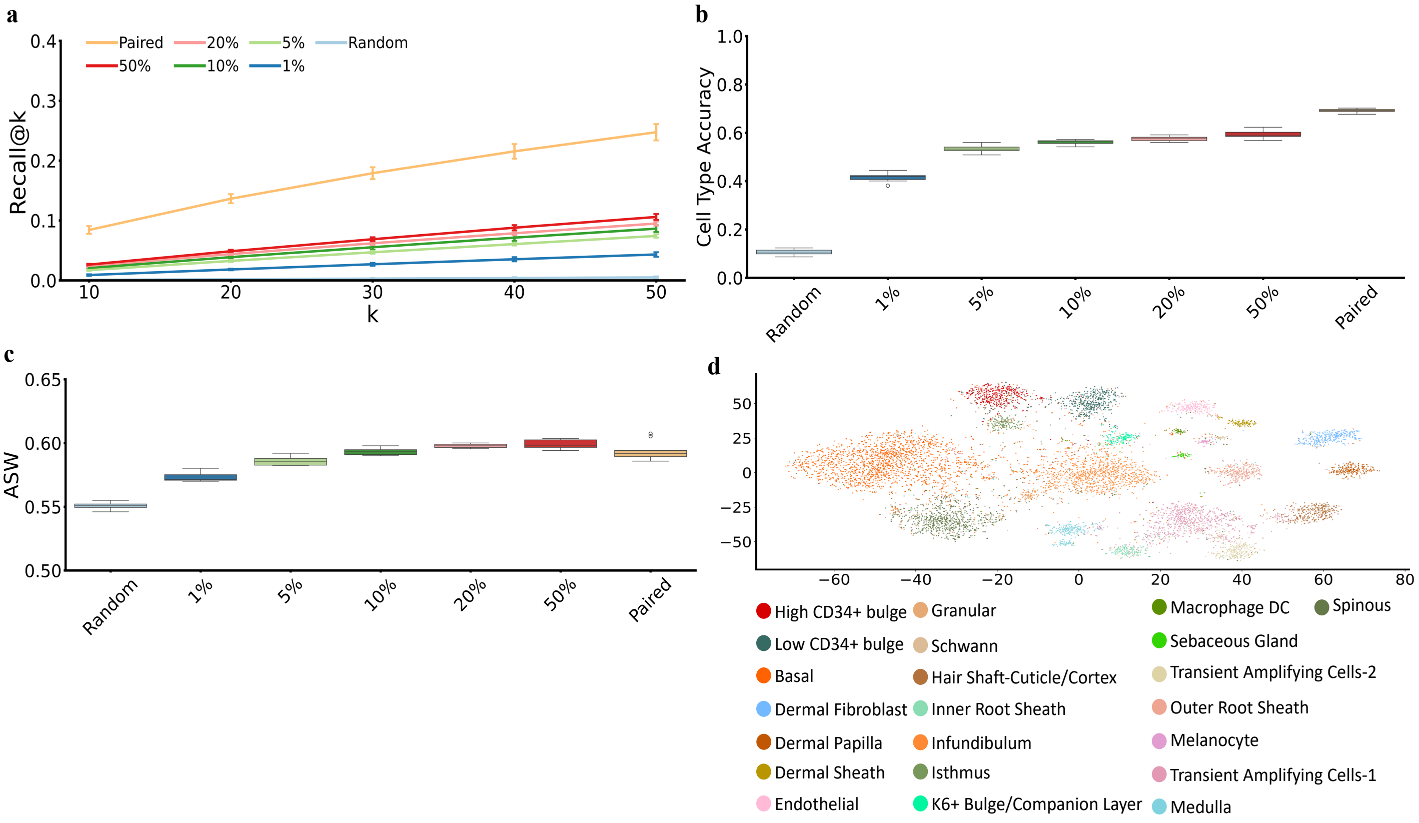

Supplementary Figure 2. Comparison of metrics across unpaired, random, and paired settings in the SHARE-seq dataset. **a)** Recall@k **b)** Cell type accuracy **c)** ASW based on the joint embeddings **d)** t-SNE representations of the embeddings from the hold-out dataset colored by cell types. delete

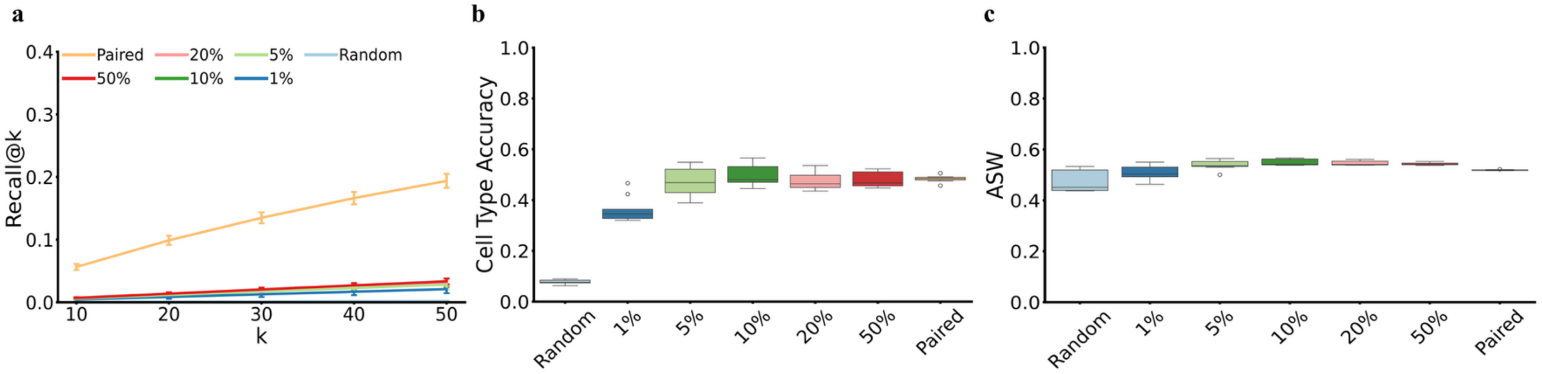

Supplementary Figure 3. Comparison of metrics across unpaired, random, and paired settings in the CITE-seq dataset. **a)** Recall@k **b)** Cell type accuracy **c)** ASW based on the joint embeddings

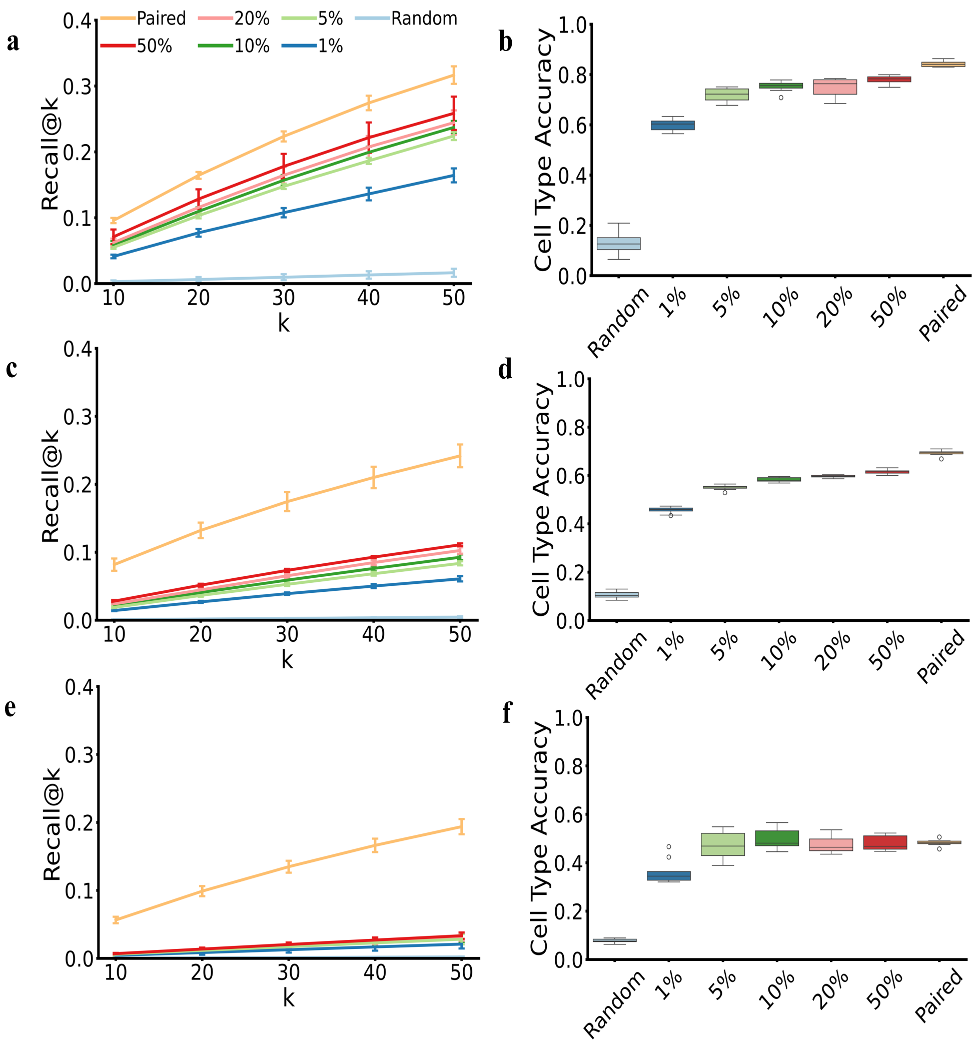

Supplementary Figure 4. Recall@k and cell type accuracy values from **a,b)** gene expression to chromatin accessibility for PBMC data, **c,d)** gene expression to chromatin accessibility for SHARE-seq data and **e,f)** gene expression to cell surface proteins for CITE-seq data.

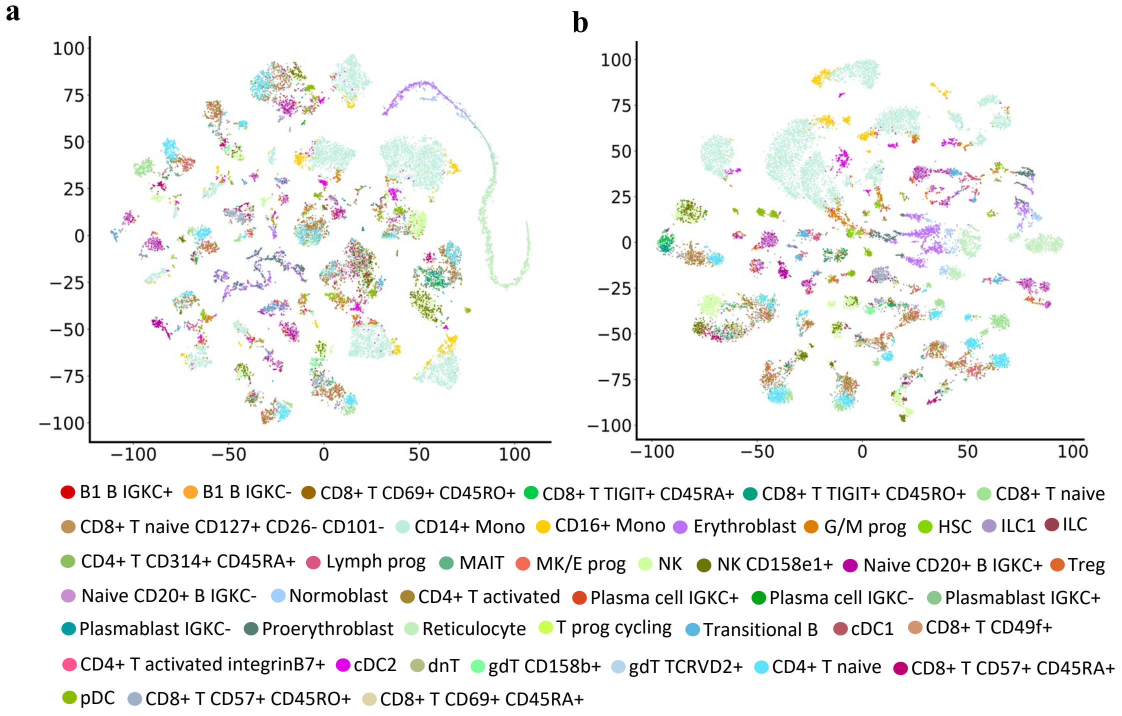

Supplementary Figure 5. a) t-SNE plot of the original data (scRNA-seq and scADT). **b)** t-SNE plot of the sCIN's integrated embeddings.

Supplementary Tables

Supplemetary Table 1. The comparison of the sCIN's strategy for selection of positive and negative pairs based on matched cells in the paired case and cell types in the unpaired case with the random selection of positive and negative pairs. This experiment performed for unseen paired datasets namely SHARE-seq (a), PBMC (b), and CITE-seq (c), and unseen unpaired Muto-2021 dataset (d). All experiments replicated 10 times and the average vlaues of metrics across replications in addition to the standard deviations are reported.

a) SHARE-seq dataset

Model	Cell type accuracy	ASW	Median rank	Recall@10	Recall@30	Recall@50
sCIN	0.69 ± 0.007	0.59 ± 0.006	334 ± 57.8	0.08 ± 0.006	0.17 ± 0.009	0.24 ± 0.013
sCIN_random_pairs	0.10 ± 0.005	0.46 ± 0.005	4861 ± 462.4	0.001± 0.0002	0.003 ± 0.0003	0.005 ± 0.0006

b) PBMC dataset

Model	Cell type accuracy	ASW	Median rank	Recall@10	Recall@30	Recall@50
sCIN	0.84 ± 0.044	0.70 ± 0.005	106 ± 2.3	0.09 ± 0.003	0.22 ± 0.006	0.31 ± 0.009
sCIN_random_pairs	0.13 ± 0.008	0.48 ± 0.005	1,405 ± 208.4	0.003± 0.001	0.011 ± 0.002	0.018 ± 0.004

c) CITE-seq dataset

Model	Cell type accuracy	ASW	Median rank	Recall@10	Recall@30	Recall@50
sCIN	0.48 ± 0.011	0.51 ± 0.001	520 ± 126.9	0.05 ± 0.004	0.13 ± 0.008	0.19 ± 0.011
sCIN_random_pairs	0.09 ± 0.022	0.32 ± 0.014	12,969 ± 1,559	0.0003± 0.0001	0.01 ± 0.0004	0.001 ± 0.0007

d) Muto-2021 dataset

Model	ASW	Cell type@10	Cell type@30	Cell type@50
sCIN	0.76 ± 0.088	0.93 ± 0.020	0.93 ± 0.022	0.93 ± 0.023
sCIN_random_pairs	0.34 ± 0.026	0.17± 0.034	0.17 ± 0.032	0.17 ± 0.039

Supplementary Table 2. sCIN's training time for paired and unpaired datasets. 'Number of cells' column contains the number of cells used for training. For the SHARE-seq, PBMC, and Muto-2021 datasets, the first and the second modality dimensions are related to gene expression and chromatin accessibility, respectively. For the CITE-seq data, the first and the second modalities are gene expression and cell surface proteins, respectively. The model was trained on one Nvidia GeForce RTX 4090 Graphical Processing Unit (GPU) in GNU/Linux (x86_64) operating system.

Data	Number of cells	Dimension (Modality 1)	Dimension (Modality 2)	Training time (min)
CITE-seq	63,182	13,953	134	43.56
SHARE-seq	22,561	21,478	340,341	129.60
Muto-2021	13,989 (Modality 1)<br>16,943 (Modality 2)	27,146	99,019	40.78
PBMC	6,741	29,095	107,194	8.66

Supplementary Table 3. The comparison of sCIN's performance with benchmark models. These experiments were performed for the unseen paired SHARE-seq dataset. All experiments replicated 10 times and we reported the average value of each metric across replications in this table.

Models	Median rank	Cell type accuracy	ASW	Recall@10	Recall@30	Recall@50
sCIN	334 ± 57.8	0.69 ± 0.007	0.59 ± 0.006	0.0842 ± 0.006	0.17 ± 0.009	0.24 ± 0.013
Con-AAE	401± 35.8	0.64 ± 0.021	0.66 ± 0.005	0.0321 ± 0.004	0.08 ± 0.008	0.12 ± 0.010
scGLUE	3,265 ± 1669.3	0.48 ± 0.105	0.49 ± 0.006	0.0214 ± 0.006	0.04 ± 0.013	0.06 ± 0.017
MOFA+	749 ± 51.4	0.59 ± 0.02	0.47 ± 0.002	0.0118 ± 0.001	0.03 ± 0.003	0.05 ± 0.004
Harmony	4,816 ± 23.5	0.10 ± 0.008	0.46 ± 0.007	0.0008 ± 0.0003	0.002 ± 0.0004	0.004 ± 0.0005
Autoencoder	4,839 ± 131.5	0.11 ± 0.012	0.47 ± 0.004	0.0009 ± 0.0002	0.002 ± 0.0006	0.004 ± 0.0008
sciCAN	5,404 ± 263.4	0.10 ± 0.009	0.49 ± 0.001	0.0009 ± 0.0003	0.002 ± 0.0006	0.004 ± 0.0009
scBridge	5,196 ± 89.6	0.09 ± 0.004	0.49 ± 0.001	0.0007 ± 0.0002	0.002 ± 0.0005	0.004 ± 0.0004

Supplementary Table 4. The comparison of sCIN's performance with benchmark models. These experiments were performed for the unseen paired PBMC dataset. All experiments replicated 10 times and we reported the average value of each metric across replications in this table.

Models	Median rank	Cell type accuracy	ASW	Recall@10	Recall@30	Recall@50
sCIN	106 ± 2.3	0.84 ± 0.008	0.87 ± 0.005	0.09 ± 0.003	0.22 ± 0.006	0.31 ± 0.009
Con-AAE	116 ± 5.8	0.81 ± 0.01	0.82 ± 0.003	0.08 ± 0.007	0.20 ± 0.023	0.29 ± 0.009
scGLUE	908 ± 108.0	0.67 ± 0.03	0.55 ± 0.004	0.02 ± 0.005	0.06 ± 0.011	0.09 ± 0.01
MOFA+	1,302 ± 80.4	0.35 ± 0.05	0.55 ± 0.007	0.005 ± 0.001	0.01 ± 0.004	0.02 ± 0.006
Harmony	1,437 ± 20.0	0.13 ± 0.002	0.48 ± 0.001	0.003 ± 0.001	0.01 ± 0.002	0.01 ± 0.002
Autoencoder	1,462 ± 51.7	0.13 ± 0.03	0.51 ± 0.004	0.002 ± 0.001	0.009 ± 0.002	0.01 ± 0.003
sciCAN	1,413 ± 53.3	0.09 ± 0.009	0.49 ± 0.002	0.003 ± 0.001	0.008 ± 0.002	0.01 ± 0.02
scBridge	222 ± 22.3	0.59 ± 0.03	0.62 ± 0.006	0.04 ± 0.005	0.11 ± 0.008	0.16 ± 0.014

Supplementary Table 5. The comparison of sCIN's performance with benchmark models. These experiments were performed for the unseen paired CITE-seq dataset. All experiments replicated 10 times and we reported the average value of each metric across replications in this table.

Models	Median rank	Cell type accuracy	ASW	Recall@10	Recall@30	Recall@50
sCIN	520 ± 126.9	0.48 ± 0.011	0.51 ± 0.001	0.05 ± 0.004	0.13 ± 0.008	0.19 ± 0.011
Con-AAE	12,784 ± 258.5	0.10 ± 0.105	0.31 ± 0.312	0.0005 ± 0.0001	0.001 ± 0.0004	0.002 ± 0.0007
scGLUE	14,234 ± 1,169.6	0.08 ± 0.008	0.26 ± 0.018	0.0003 ± 0.000	0.0008 ± 0.0002	0.0015 ± 0.0004
MOFA+	12,274 ± 281.8	0.11 ± 0.007	0.39 ± 0.005	0.001 ± 0.0002	0.004 ± 0.0004	0.007 ± 0.0005
Harmony	13,773 ± 85.9	0.08 ± 0.013	0.46 ± 0.004	0.0002 ± 0.000	0.0008 ± 0.0002	0.0015 ± 0.0004
Autoencoder	13,436 ± 160.7	0.08 ± 0.020	0.35 ± 0.037	0.0004 ± 0.000	0.0011 ± 0.0002	0.002 ± 0.0004
sciCAN	13,508 ± 375.5	0.09 ± 0.012	0.45 ± 0.010	0.0004 ± 0.0001	0.0013 ± 0.0004	0.0022 ± 0.0007

Supplementary Table 6. The comparison of sCIN's performance with benchmark models. These experiments were performed for the unseen unpaired Muto-2021 dataset. All experiments replicated 10 times and we reported the average value of each metric across replications in this table.

Models	ASW	Cell type@10	Cell type@30	Cell type@50
sCIN	0.76 ± 0.088	0.94 ± 0.020	0.93 ± 0.022	0.93 ± 0.023
scGLUE	0.52 ± 0.017	0.56 ± 0.174	0.56 ± 0.172	0.56 ± 0.167
sciCAN	0.48 ± 0.0005	0.87 ± 0.002	0.78 ± 0.004	0.71 ± 0.004
MOFA+	0.56 ± 0.002	0.19 ± 0.026	0.21 ± 0.02	0.21 ± 0.02
